# Drug-induced Reduction of Gamma Carboxylation in Osteocalcin: What is the Fallback?

**DOI:** 10.7759/cureus.5504

**Published:** 2019-08-28

**Authors:** Norina Usman, Aisha Qaseem, Joseph S Jayaraj, Nida Fathima, Rajesh Naidu Janapala

**Affiliations:** 1 Internal Medicine, Veterans Affairs Palo Alto Health Care System - Stanford University School of Medicine, Palo Alto, USA; 2 Internal Medicine, California Institute of Behavioral Neurosciences and Psychology, Fairfield, USA; 3 Internal Medicine, Icahn School of Medicine at Mount Sinai/Queens Hospital Center, New York, USA; 4 Internal Medicine, Sri Siddhartha Medical College, Tumkur, IND

**Keywords:** osteoporosis, fracture, warfarin, anticoagulants

## Abstract

Osteoporosis is a medical condition in which bone becomes fragile and weak. In this condition, the quality and density of the bone are reduced. Vitamin K is vital for bone mineralization as it plays a vital role in the gamma-carboxylation of osteocalcin. Therefore, if there is a deficiency of vitamin K, it can lead to osteoporosis due to undercarboxylated osteocalcin.

Warfarin is the most widely used anticoagulant in the elderly. In this article, we reviewed how Warfarin, an inhibitor of vitamin K, affects bone remodeling and leads to osteoporosis

## Introduction and background

Warfarin is a traditional oral anticoagulant used in elderly patients for stroke prevention with non-valvular atrial fibrillation (NVAF). It is used in many other diseases such as myocardial infarction, deep vein thrombosis (DVT), and pulmonary embolism (Table 1). It has many side effects, among which hemorrhage is the most common; other side effects are purple toe syndrome, warfarin necrosis, osteoporosis, and calcification. Warfarin is a vitamin K antagonist that interferes with the gamma-carboxylation of glutamic acid residues. Therefore, it inhibits the activation of bone matrix proteins [1]. Warfarin effect should be monitored every one to four weeks by assessing the prothrombin time/International Normalized Ratio (INR).

**Table 1:** Uses of warfarin

1. Long-term prophylaxis of thrombosis
Prevention of thrombosis during atrial fibrillation
Prevention of thromboembolism (in patients with prosthetic heart valves)
Prevention of venous thrombosis and associated pulmonary embolism
Treatment of Myocardial infarction and stroke
2. Not useful in emergencies

Several analyses have reported an amplified risk of osteoporotic fractures with the use of Warfarin [1-4]. This concern was confirmed by a population-based study of 14,564 Medicare patients in the United States in 2006, which highlighted a higher risk of osteoporotic fracture in patients diagnosed with Atrial Fibrillation (AF) (odds ratio: 1.25) and long-term (one year) warfarin users as compared with Warfarin non-users [3].

Two hundred fifty thousand people in the United States are hospitalized with hip fractures per year [5]. All patients admitted with these fractures, between 7.8% and 10.3% are receiving chronic warfarin therapy [6-9]. In the coagulation cascade, vitamin K acts as a necessary cofactor in the gamma-carboxylation of glutamate to γ-carboxyglutamate (Gla) residues in many coagulation proteins (proteins C and S) and clotting factors (factors II, VII, IX, and X). By inhibiting vitamin K, coagulation is also inhibited. Warfarin antagonizes vitamin K-dependent processes, including the gamma-carboxylation of osteocalcin and bone matrix proteins such as Gla proteins that are required in bone mineralization (Table 2). Previous studies have shown that there is an association between the augmented level of undercarboxylated osteocalcin in warfarin users and amplified fracture risk, caused due to reduced bone mineral density. Osteocalcin in the carboxylate form binds to calcium and then to hydroxyl-apatite crystals in the matrix of the bone [10-12].

**Table 2:** Vitamin K-dependent proteins GAS6 - Growth Arrest Specific 6

NAME	TISSUE	FUNCTION
Osteocalcin (OC)	Bone	Contribute to bone health
Matrix Gla Protein (MGP)	Aorta, heart valve	Inhibit calcification in arteries
Coagulation factors/Anti-coagulation factors	Liver	Contribute to normal coagulation
GAS6	Aorta, brain	Modulate cell growth

If Vitamin K is low, it will lead to a low level of undercarboxylated osteocalcin, which increases the fracture risk in the elderly (Tables 3-4) [13-14]. Further, the serum concentration of undercarboxylated osteocalcin has been revealed to increase with warfarin [15-16]. Despite the fact that warfarin increases fracture risk, it was an unavoidable treatment choice for years because no other comparable alternatives were available. For the prevention of atrial fibrillation and treatment of medical conditions such as acute coronary syndromes, venous thromboembolism. Several alternate options for warfarin are being investigated including oral direct thrombin inhibitors (Table 5). A new oral direct thrombin inhibitor Dabigatran has been approved for stroke prevention in patients diagnosed with atrial fibrillation. Dabigatran has a better basal characteristic profile as compared to warfarin (Table 5). Unlike warfarin, Dabigatran is being administered by a fixed-dose without any need for dosing adjustments and routine coagulation monitoring [17]. In this article, we tried to find an alternative coagulation therapy (as opposed to traditional warfarin therapy), as a means to avoid the risk of osteoporosis associated with warfarin. 

**Table 3:** Sources of vitamin K

Alfa alfa
Cabbage
Cauliflower
Cheese
Dairy products
Egg yolk
Green leafy
Liver
Meat
Tomato
Spinach
Vegetables

**Table 4:** Function of vitamin K

Promotes bone calcification
Prevents blood vessel calcification
Assists in blood clotting

**Table 5:** Pharmacokinetics and pharmacodynamics of newer anticoagulants

	Apixaban	Rivaroxaban	Edoxaban	Dabigatran
Mode of Excretion	75% feces 25% renal	66% renal 34% feces	50% renal 50% feces	80% renal 20% fecal
Max Concentration	3 hours	2-4 hours	1-2 hours	1 hour
Dosing	Twice a day	Daily	Daily	Twice a day
Half-life	approximately 12 hours	Healthy Patients: 5-9 hours, Elderly Patients: 9-13 hours	10-14 hours	12-17 hours
Cascade Target	Factor Xa	Factor Xa	Factor Xa	Factor lla

## Review

Mechanism of osteoporosis by warfarin

**Figure 1:**
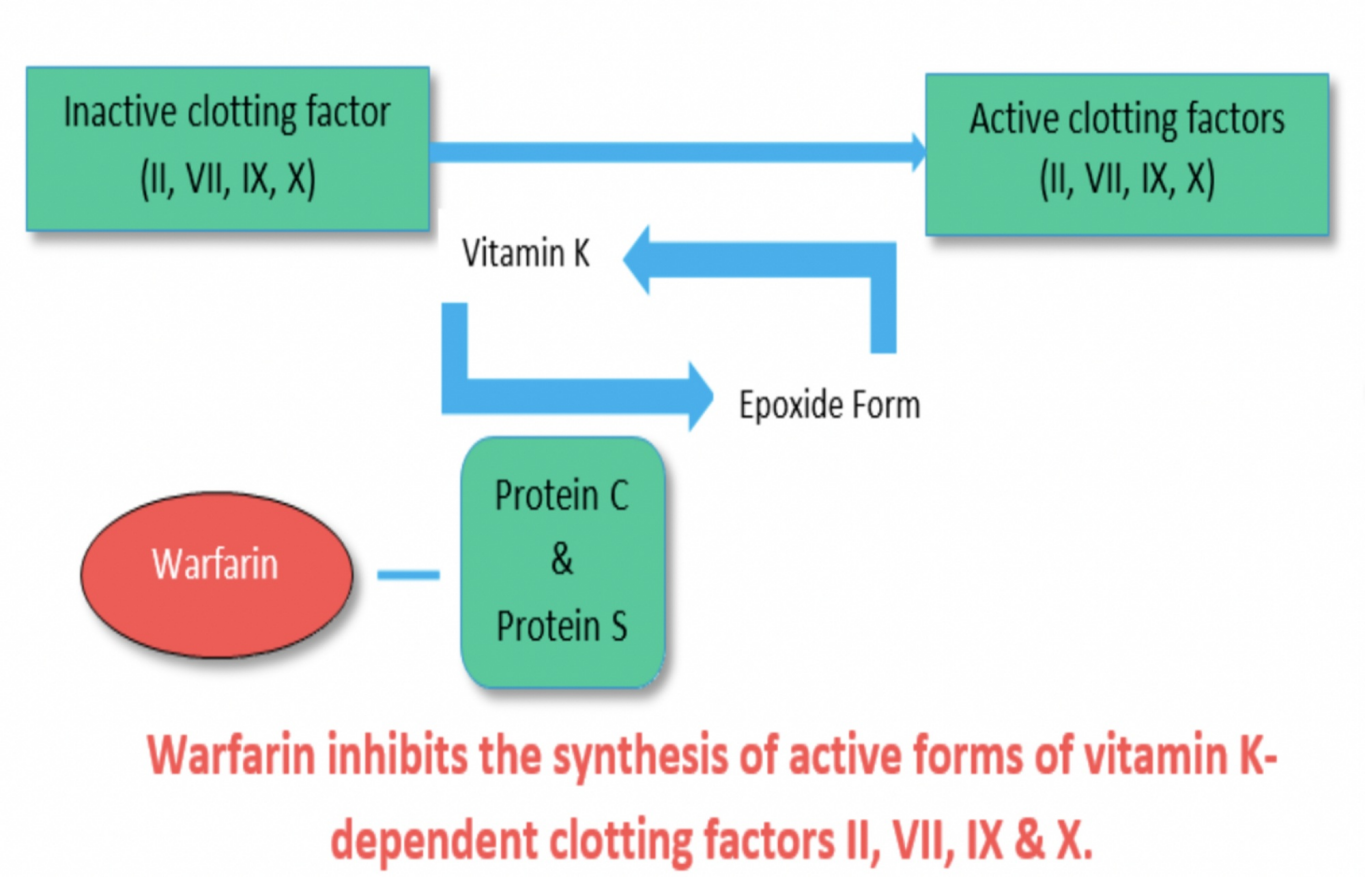
Warfarin’s mechanism of action

Osteoporosis is an illness in which bone loss is more than bone formation leading to the weak and brittle bones in the older age group. The bone becomes so soft that even minor stress can lead to fractures. The most common bones involved are vertebrae, spine, hip, and forearm. The symptom does not appear until the bone is fractured, and the most common symptom is back pain, short height, and stooped posture. In 1970, the sequence of amino acid in the bone G1a protein was determined, which is known as osteocalcin. It was confirmed that it plays a major role in bone mineralization [18]. The unique structure of G1a protein allows the binding of calcium ions and calcium mineral surface at position 17, 21, and 24 with three gamma-carboxyglutamic acid residues [19]. Vitamin K is a cofactor in the gamma-carboxylation of osteocalcin that helps in bone mineralization. If vitamin K is low, it can increase the risk of osteoporosis. High levels of y-carboxylation make strong bones, and low-levels lead to more bone loss. It has been reported that undercarboxylated osteocalcin (uOC) increases after menopause and much higher after the age of 70, which causes a decrease in BMD and an increase in bone loss, resulting in hip fracture. Warfarin inhibits the vitamin-K dependent y-carboxylation of osteocalcin, leading to osteoporosis, through two ways, directly and indirectly: (1) Directly by inhibiting gamma-carboxylation of osteocalcin and (2) indirectly because patients on Warfarin are recommended to take diet low in vitamin-k (Figure 1). 

Warfarin increases skeletal fragility by decreasing bone mineral density and inducing the risk of vertebral and rib fractures through vitamin K deficiency. There is some controversy that there is warfarin-induced impairment of bone quality rather than quantity and also depends on the period of treatment, as one of the studies showed that there was no amplified osteoporotic fracture risk in patients who were using Warfarin for less than one year [19-21]

Warfarin versus dabigatran

**Figure 2:**
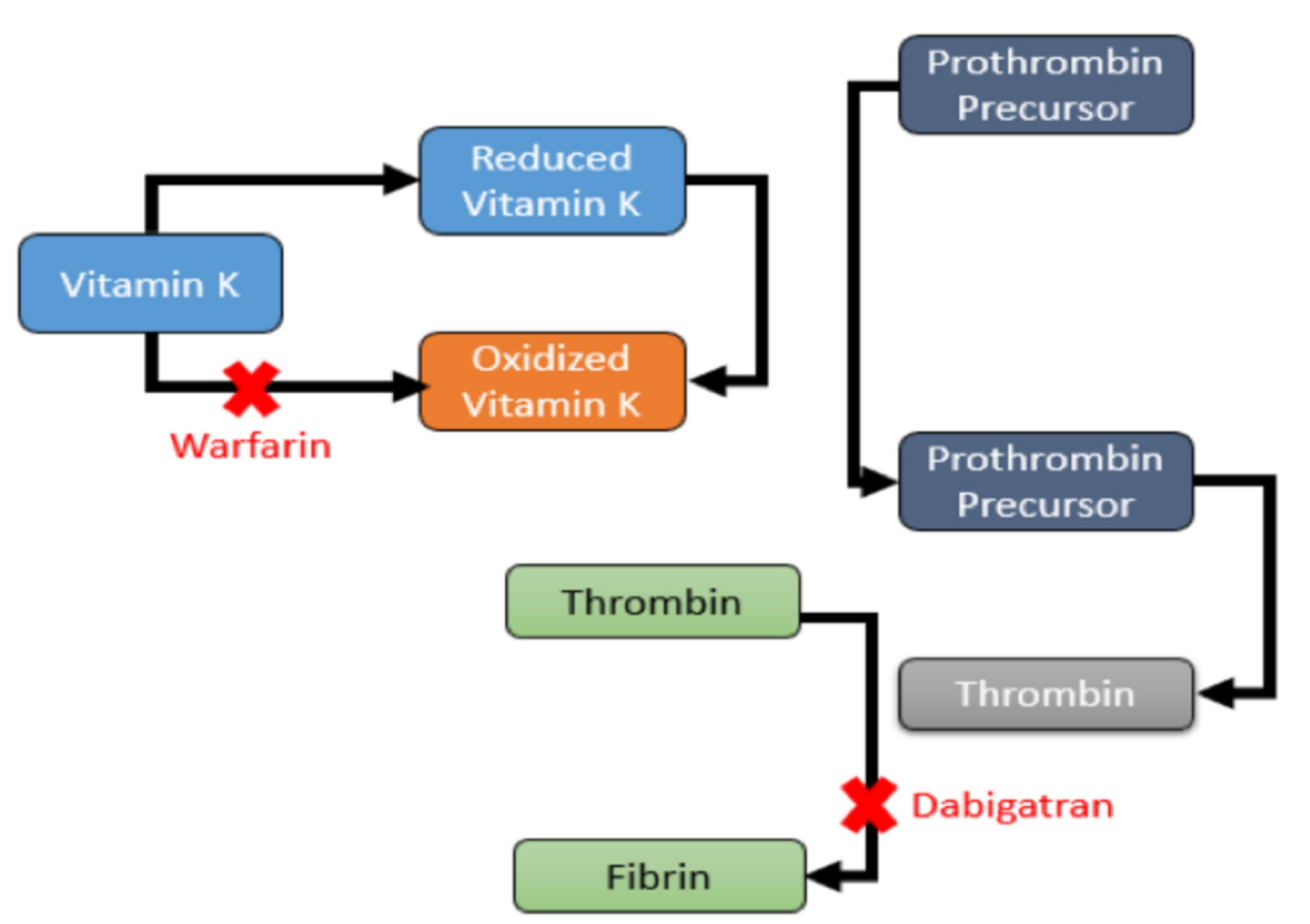
Comparison of the mechanism of action between warfarin and Dabigatran

An oral anticoagulant ethoxylate dabigatran is a pro-drug that is converted by serum esterase to more potent form, Dabigatran directly inhibits thrombin by preventing the conversion of fibrinogen into fibrin (Figure 2). Dabigatran has a half-life of 12 to 17 hours and bioavailability of 6.5%. Dabigatran does not need routine monitoring as compared to warfarin [22]. In a pilot trial that involved patients with atrial fibrillation. Dabigatran has been given for the prevention of venous thromboembolism, doses of 220 mg once daily, and 150 mg twice daily, were promising [23-24]. Another large randomized trial also compared the same treatments of dabigatran with warfarin in patients who were diagnosed with atrial fibrillation, and the result was very assuring. Lower rates of hemorrhage and comparable rates of systemic embolism and stroke were associated with the 110-mg dose of dabigatran when compared to Warfarin; A similar rate of major hemorrhage and lower rates of systemic embolism and stroke was associated with a 150-mg dose of dabigatran (Table 6).

**Table 6:** Comparison of basal characteristics between warfarin and dabigatran AF, atrial fibrillation

Property	Warfarin	Dabigatran
Mechanism of action	Reduced synthesis of prothrombin and other clotting factors	Direct inhibition of thrombin
Onset of action Elimination half-life Duration of action	36-72 hours 20-60 hours 48-96 hours	0.5-2 hours 12-14 hours 24 hours
Indication for AF	Valvular or non-valvular atrial fibrillation	Non-valvular atrial fibrillation
Dosing	Individualized to each patient and target INR	Fixed-dose, dependent on creatinine clearance and age
Administration	Oral Once daily	Oral Twice Daily (for AF)

As compared to warfarin, dabigatran is not vitamin K-dependent anticoagulant, which is approved to be used in patients with NVAF. In recent animal studies, it was reported that the application of dabigatran lowers bone turn over, increases bone volume leading to a low risk of osteoporotic fracture as compared to Warfarin. There was another in vivo study done recently, which indicated less trabecular separation, higher bone volume compared with warfarin use in rats, but no similar study was conducted in humans. In this study, they compared the effect of warfarin and dabigatran on bone structure in rats without any renal impairment. Histomorphometry study was done, and the results indicated that there was increased Warfarin mediated osteoclastic activity leading to a significant decrease in bone volume and an increase in trabecular separation which determines alteration in bone quality and high risk of fractures. As compared to warfarin, dabigatran-treated group was safe as it lacks the increased osteoclastic activity and any structural impairment, being more reliable in this context. However, after chronic use of warfarin, which is associated with the amplified osteoclastic activity, a significant reduction in bone volume and increases in trabecular separation might establish a higher propensity to fractures and alterations in bone quality [25]. Therefore, the amplified risk of warfarin-induced fracture in patients is because of quality, not the quantity of bone, also based on the period of the use. Biologically, dabigatran is vitamin- k independent anticoagulant, other than that patients are not asked to reduce vitamin K in the diet; this could be another reason that Dabigatran is not causing osteoporosis. These studies have claimed that dabigatran is an excellent alternative to warfarin if we want to avoid osteoporotic fractures. 

## Conclusions

The patients needing anticoagulants such as warfarin are often at risk for osteoporosis. This happens because the patients on Warfarin are suggested to be on a vitamin K-free diet. However, vitamin K is necessary for bone mineralization. This review suggests that the anticoagulant Dabigatran is relatively safe in comparison to warfarin - as it is vitamin K independent - and does not require any dietary restrictions of vitamin K. However, the data currently present on this subject is not sufficient. From this article, the questions that arise are “if warfarin use induces osteoporosis, why don’t all patients on warfarin suffer from osteoporosis?”, “what are the factors that protect warfarin users from developing osteoporosis?” and “what are the factors that limit the transition of warfarin to dabigatran in routine medical practice?”. Therefore, we recommend more studies in the near future on this subject to find more treatment options for the patients already who have osteoporosis needing anti-coagulants.

## References

[1] Sugiyama T, Kugimiya F, Kono S (2015). Warfarin use and fracture risk: an evidence-based mechanistic insight. Osteoporos Int.

[2] Caraballo PJ, Heit JA, Atkinson EJ (1999). Long-term use of oral anticoagulants and the risk of fracture. Arch Intern Med.

[3] Gage BF, Birman-Deych E, Radford MJ (2006). Risk of osteoporotic fracture in elderly patients taking warfarin: results from the National Registry of Atrial Fibrillation 2. Arch Intern Med.

[4] Rejnmark L, Vestergaard P, Mosekilde L (2007). Fracture risk in users of oral anticoagulants: a nationwide case-control study. Int J Cardiol.

[5] Hirsh J, Dalen J, Anderson DR (2001). Oral anticoagulants: mechanism of action, clinical effectiveness, and optimal therapeutic range. Chest.

[6] (2016). National Hospital Discharge Survey. http://www.cdc.gov/nchs/nhds/index.htm.

[7] Eardley WG, Macleod KE, Freeman H (2014). "Tiers of delay": warfarin, hip fractures, and target-driven care. Geriatr Orthop Surg Rehabil.

[8] Gleason LJ, Mendelson DA, Kates SL (2014). Anticoagulation management in individuals with hip fracture. J Am Geriatr Soc.

[9] Tran T, Delluc A, de Wit C (2015). The impact of oral anticoagulation on time to surgery in patients hospitalized with hip fracture. Thromb Res.

[10] Rubinacci A (2009). Expanding the functional spectrum of vitamin K in bone. Focus on: “Vitamin K promotes mineralization, osteoblast to osteocyte transition, and an anti-catabolic phenotype by γ-carboxylation-dependent and -independent mechanisms”. Am J Physiol Cell Physiol.

[11] Price PA (1985). Vitamin K-dependent formation of bone Gla protein (osteocalcin) and its function. Vitam Horm.

[12] Gundberg CM, Lian JB, Booth SL (2012). Vitamin K-dependent carboxylation of osteocalcin: friend or foe?. Adv Nutr.

[13] Szulc P, Chapuy MC, Meunier PJ (1996). Serum undercarboxylated osteocalcin is a marker of the risk of hip fracture: a three-year follow-up study. Bone.

[14] Luukinen H, Herala M, Koski K (2000). Fracture risk associated with a fall according to the type of fall among the elderly. Osteoporos Int.

[15] Knapen MH, Hamulyak K, Vermeer C (1989). The effect of vitamin K supplementation on circulating osteocalcin (bone Gla protein) and urinary calcium excretion. Ann Intern Med.

[16] Haffa A, Krueger D, Bruner J (2000). Diet- or warfarin-induced vitamin K insufficiency elevates circulating undercarboxylated osteocalcin without altering skeletal status in growing female rats. J Bone Miner Res.

[17] Greig SL, McKeage K (2014). Dabigatran etexilate: a review of its use in the treatment of acute venous thromboembolism and prevention of venous thromboembolism recurrence. Drugs.

[18] Price Price, PA PA, Poser Poser, JW JW, Raman Raman (1976). Primary structure of the gamma-carboxyglutamic acid-containing protein from bovine bone. Proc Natl Acad Sci U S A.

[19] Hauschka PV, Carr SA (1982 ). Calcium-dependent alpha-helical structure in osteocalcin. Biochemistry.

[20] Philip WJ, Martin JC, Richardson JM (1995). Decreased axial and peripheral bone density in patients taking long-term warfarin. QJM.

[21] Caraballo PJ, Gabriel SE, Castro MR (1999). Changes in bone density after exposure to oral anticoagulants: a meta-analysis. Osteoporos Int.

[22] Stangier J (2008). Clinical pharmacokinetics and pharmacodynamics of the oral direct thrombin inhibitor dabigatran etexilate. Clin Pharmacokinet.

[23] Ezekowitz MD, Reilly PA, Nehmiz G (2007). Dabigatran with or without concomitant aspirin compared with warfarin alone in patients with nonvalvular atrial fibrillation (PETRO Study). Am J Cardiol.

[24] Eriksson BI, Dahl OE, Rosencher N (2007). Dabigatran etexilate versus enoxaparin for prevention of venous thromboembolism after total hip replacement: a randomized, double-blind, non-inferiority trial. Lancet.

[25] Fusaro M, Dalle Carbonare L, Dusso A (2015). Differential effects of dabigatran and warfarin on bone volume and structure in rats with normal renal function. PLoS One.

